# Dance training improves the CNS’s ability to utilize the redundant degrees of freedom of the whole body

**DOI:** 10.1038/s41598-020-79081-9

**Published:** 2020-12-17

**Authors:** Kyung Koh, Yang Sun Park, Da Won Park, Jae Kun Shim

**Affiliations:** 1 Department of Kinesiology, University of Maryland, College Park, MD USA; 2 Department of Sports Welfare, Korea National University of Transportation, Chungcheongbuk-do, South Korea; 3 Department of Kinesiology, Seoul National University, Seoul, South Korea; 4 Department of Mechanical Engineering, Kyung Hee University, Yongin-Si, Gyeonggi-do South Korea; 5 Neuroscience and Cognitive Science Program, University of Maryland, College Park, MD USA; 6 Fischell Department of Bioengineering, University of Maryland, College Park, MD USA; 7 Present Address: Department of Physical Therapy and Rehabilitation Science, University of Maryland, Baltimore, MD USA

**Keywords:** Neuroscience, Motor control

## Abstract

Professional dancers demonstrate an amazing ability to control their balance. However, little is known about how they coordinate their body segments for such superior control. In this study, we investigated how dancers coordinate body segments when a physical perturbation is given to their body. A custom-made machine was used to provide a short pulling impulse at the waist in the anterior direction to ten dancers and ten non-dancers. We used Uncontrolled Manifold analysis to quantify the variability in the task-relevant space and task-irrelevant space within the multi-dimensional space made up of individual segments’ centers of mass with a velocity adjustment. The dancers demonstrated greater utilization of redundant degrees of freedom (DoFs) supported by the greater task-irrelevant variability as compared to non-dancers. These findings suggest that long-term specialized dance training can improve the central nervous system’s ability to utilize the redundant DoFs in the whole-body system.

## Introduction

Humans are capable of robust balance control despite many challenges caused by external perturbations^1^ as well as internal ones^2^. To achieve this remarkable balance control, the central nervous system (CNS) is required to coordinate multiple body segments for the stabilization of the overall action of the whole body. The concept of motor synergies has been proposed as a control mechanism of the CNS^3^, which refers to task-specific patterns of the multi-degrees of freedoms (DoFs) that stabilize the performance of a particular motor task^4^. Cumulating evidence on postural control has suggested that the CNS is able to synergistically control multiple DoFs such as multiple segments, multiple muscles, and multiple fingers for the stabilization of their combined actions such as whole body center of mass^5,6^, overall muscle activations^7,8^, and multi-finger grasping^9,10^.

Motor synergy has often been quantified using the Uncontrolled Manifold (UCM) approach^11,12^ in a variety of motor tasks. The UCM approach provides an analysis that allows to quantify how DoFs are organized or coordinated through the structure of variability of DoFs with respect to a given task performance. For instance, in an arm-reaching task, there are an infinite number of joint configurations that can result in an equivalent fingertip position. This is possible because there exist more DoFs in the arm than those that are strictly required for the particular motor task, and this phenomenon is known as the problem of motor redundancy^13^. There are two types of variability in a redundant motor system: variability that directly affects the performance of a motor task (i.e. task-relevant variability) and variability that does not influence motor performance (i.e. task-irrelevant variability). While task-relevant variability may be considered error associated with a particular motor task, task-irrelevant variability is regarded as reflection of solutions adapted by the CNS. The ratio of task-irrelevant variability (i.e. “good” variability) to task-relevant variability (i.e. “bad” variability) has been often computed as an index of motor synergy in a redundant motor system^10–12^.

Utilizing the UCM approach, previous studies on similarities and differences of motor synergies in different populations have improved our understanding of how the CNS controls a redundant motor system. In healthy populations, many studies found that task-irrelevant variability was greater than task-relevant variability, indicating more flexibility in utilizing DoFs to accomplish a task. Such flexibility has been considered beneficial in dealing with changes in external and internal constraints, such as unexpected perturbations^14^, fatigue^15^, and secondary tasks^16^. On the other hand, in pathological and gerontological groups, different patterns of motor synergies have been observed often with decreased task-irrelevant variability and increased task-relevant variability as compared to healthy and young groups. The abnormal motor synergies have been suggested as pathological and gerontological markers of deficits or changes in the CNS control mechanisms for dealing with a redundant motor system. Similarly, studies of professional dancers and athletes with years of specialized training in whole body balance and coordination may provide insight into CNS control mechanisms. Specifically, dancers have an amazing ability to control their postural balance gained through specialized training^17^. Thus, their superior abilities of postural control may be reflected in motor synergies. Although there has been a great deal of research exploring expert performance of motor tasks^18,19^, it is largely unknown how dancers coordinate their body segments for the stabilization of the whole-body action.

Dynamic postural control is often defined as the ability to control our body in a way to maintain or return the whole-body center of mass (CoM) over its base of support (BoS)^20^. Whole-body CoM is commonly calculated as weighted sum of individual CoMs. Although this quantification of the whole-body CoM may be used in quasi-static motor tasks such as quiet standing tasks for evaluation of postural control^21,22^, it may not be appropriate for more dynamic motor tasks involving significant magnitudes of the whole-body CoM velocity. Due to this reason, the whole-body CoM in dynamic postural control has often been quantified using a concept known as the ‘extrapolated center of mass’ (xCoM)^23–25^. The xCoM is a velocity-adjusted CoM that is derived from a linearized inverted pendulum model^26^ where the natural frequency of the motion is approximately equal to the square root of the gravitational constant over the pendulum length. Here, we investigate to what extent dancers exhibit superior ability to control multiple segments during two conditions induced by a mechanical perturbation through a waste pull for two sequential phases: (1) more stable condition where the xCoM is inside of the BoS and (2) less stable condition where the xCoM is outside the BoS. In this study, we used the UCM analysis to quantify the variability in the task-relevant space and task-irrelevant space within the multi-dimensional space made up of individual segments’ xCoMs. Professional dances train themselves for resilience in dynamic postural control in challenging conditions. Thus, we expected that dancers would demonstrate superior postural control, especially in the unstable condition by (1) showing smaller task-relevant variability and (2) greater task-irrelevant variability, as compared to non-dancers.

## Methods

### Participants

Ten female dancers (age: 27 ± 1.89 years; height: 161.70 ± 2.95 cm; weight: 49.85 ± 3.20 kg) and ten female non-dancers (age: 23.8 ± 3.79 years, height: 161.41 ± 3.23 cm; weight: 51.11 ± 4.13 kg) participated in the study. Dancers had professional dance training for 15.8 ± 4.26 years while non-dancers did not have formal dance training. None of the participants reported a history of vestibular or lower limb orthopedic injuries in a year prior to the data collection. The study was carried out in accordance with the approved guidelines and approved by the Ethics Committee of Hanyang University. A written informed consent was obtained from all participants. All methods were carried out in accordance with relevant guidelines and regulations.

### Experimental procedures

**Figure 1:**
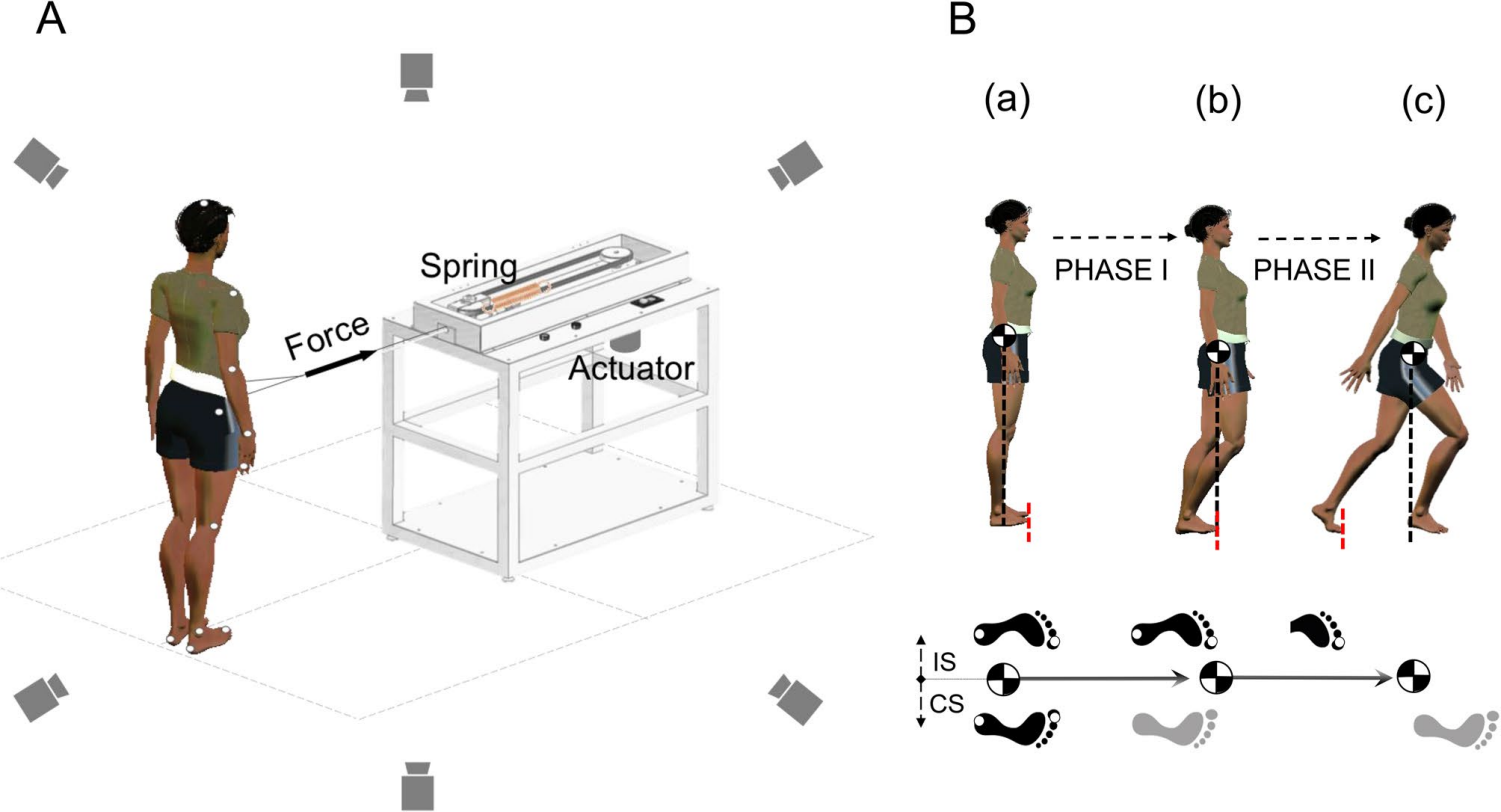
Schematic representation of experimental settings with a waist-pull apparatus and six infrared cameras. Perturbation of standing posture was provided in the anterior direction by pulling a cable connected to the subject’s belt (**A**). The stepping movement was analyzed in two phases (**B**): Phase I was defined as the period from perturbation onset (a) to the time when the $x\mathrm{CoM}$ passed outside of the BoS of the contralateral side (CS), shown in black (b). Phase II was defined as the time from (b) to foot–ground contact of ipsilateral side (IS), shown in gray (c). Drawn using Adobe Illustrator CS6 (www.adobe.com) and Microsoft Office 2016 (www.microsoft.com).

Nineteen reflective markers were placed bilaterally on the acromion process, lateral elbow, lateral wrist, end-point of the third finger, greater trochanter, lateral epicondyle of the femur, lateral malleolus, heel, toe, and head vertex. Each participant stood barefoot with a hip-width stance in front of a custom-made pulling apparatus (Fig. 1A) while wearing a belt around the waist connected to the pulling apparatus. Participants were instructed to relax and react naturally in response to the pull generated by the pulling apparatus at a random time over a 5-s period. The motor inside the pulling apparatus caused a pulling impulse to the subject’s belt. In order to provide a pulling impulse that can pose a challenge in postural control, we tried different springs with different stiffnesses and identified the spring of 20 N/cm stiffness which induced only one forward step, but not two steps, after a pull in all participants. Once the spring for follow-up experiments was identified, each participant was pulled by the pulling apparatus with the particular spring and their whole-body kinematics were recorded using a motion capture system with six infrared cameras (Visol Inc., South Korea) and Kwon3d XP software (Visol Inc., South Korea) at a sampling frequency of 100 Hz. Data analysis was performed using customized MATLAB codes (Mathworks, Natick, MA, USA).

### Variable calculations in MATLAB

#### Extrapolated center of mass

In order to estimate the whole-body CoM position in the dynamic state, we adapted the extrapolated center of mass ($x\mathrm{CoM}$) concept^26^. The xCoM is a velocity-adjusted CoM that is derived from a linearized inverted pendulum model^26^, expressed as the sum of the whole-body CoM position and its velocity scaled using the angular eigenfrequency of a non-inverted pendulum as follows (Eq. 1):

1 $$x\mathrm{CoM}=CoM+\frac {C\dot{o}M}{{w}_{o}}$$

where $CoM$ and $\dot{CoM}$ are the whole-body CoM position and velocity, respectively, and ${w}_{o}$ is the angular eigenfrequency of a non-inverted pendulum calculated as (Eq. 2):

2 $$w_{o} = \sqrt{ \frac{g}{l}}$$

where $g$ is gravitational acceleration, and $l$ is the pendulum length, computed as the distance from the lateral malleolus to the CoM of the whole-body in this study.

Whole-body $CoM$ (position) and $\dot{CoM}$ (velocity) are the weighted sum of CoM positions (${\varvec{i}}{\varvec{C}}{\varvec{o}}{\varvec{M}}:\mathrm{a}\space\mathrm{14}\space\mathrm{by}\space\mathrm{2}\space\mathrm{matrix})$ and velocities ($\dot{{\varvec{i}}{\varvec{C}}{\varvec{o}}{\varvec{M}}}:\mathrm{a}\space\mathrm{14}\space\mathrm{by}\space\mathrm{2}\space\mathrm{matrix})$ of 14 individual body segments (i.e. head, trunk, and left and right upper arms, left and right forearms, left and right hands, left and right thighs, left and right shanks, and left and right feet) calculated from the marker positions in the anterior–posterior (AP) (Eq. 3) and medial–lateral (ML) directions (Eq. 4).

3 $$CoM=\mathbf{A}\cdot {\varvec{i}}{\varvec{C}}{\varvec{o}}{\varvec{M}}$$

and

4 $$\dot{CoM}=\mathbf{A}\cdot {\varvec{i}}\dot{{\varvec{C}}{\varvec{o}}{\varvec{M}}}$$

where **A** is a 2 by 14 matrix, containing ${m}_{i}/M$ of the *i*^th^ component, $M$ is the whole-body mass, and ${m}_{i}$ is the *i*^th^ segmental mass. Fourteen individual CoM positions for individual segments were estimated using anthropometric data^27^. By replacing $CoM$ and $\dot{CoM}$ with $\mathbf{A}\cdot {\varvec{i}}{\varvec{C}}{\varvec{o}}{\varvec{M}}$ and $\mathbf{A}\cdot \mathbf{A}\cdot {\varvec{i}}\dot{{\varvec{C}}{\varvec{o}}{\varvec{M}}}$, respectively, $x\mathrm{CoM}$ is express as follows (Eq. 5):

5 $$x\mathrm{CoM}\left[t\right]=\mathbf{A}\cdot {\varvec{i}}{\varvec{x}}\mathbf{C}\mathbf{o}\mathbf{M}\left[t\right]$$

where ${\varvec{i}}{\varvec{x}}\mathbf{C}\mathbf{o}\mathbf{M}\left[t\right]={\varvec{i}}{\varvec{C}}{\varvec{o}}{\varvec{M}}+\dot{{\varvec{i}}{\varvec{C}}{\varvec{o}}{\varvec{M}}}/{w}_{o}$ , which is individual segmental extrapolated CoMs. Note that ${\varvec{i}}{\varvec{x}}\mathbf{C}\mathbf{o}\mathbf{M}\left[t\right]$ is a vector subtracted by ${\varvec{i}}{\varvec{x}}\mathbf{C}\mathbf{o}\mathbf{M}\left[0\right]$ at the onset of perturbation (t = 0).

#### Uncontrolled manifold (UCM) analysis

The uncontrolled manifold (UCM) approach has been used to analyze synergistic patterns in elemental variables for the stability of a performance variable^28^. Using the UCM analysis, we examined how multi-segments (i.e. the elemental variables) interact with each other to stabilize the actions of the whole-body CoM (i.e. the performance variable). The UCM analysis requires the use of independent elemental variables. In order to extract independent variables from individual segmental extrapolated CoMs, relative ${ix\mathrm{CoM}}_{i}$ (${iRx\mathrm{CoM}}_{i}$) at *i*th segment as ${ix\mathrm{CoM}}_{i}\left[t\right]$ subtracted from proximal segment ${ix\mathrm{CoM}}_{i-1}\left[t\right]$ was introduced. ${ix\mathrm{CoM}}_{i}\left[t\right]$ is expressed in terms of ${iRx\mathrm{CoM}}_{i}$ as follows (Eq. 6):

6 $${ix\mathrm{CoM}}_{i}\left[t\right]=\sum_{j=trunk}^{i}{iRx\mathrm{CoM}}_{j}\left[t\right]$$

**Figure 2:**
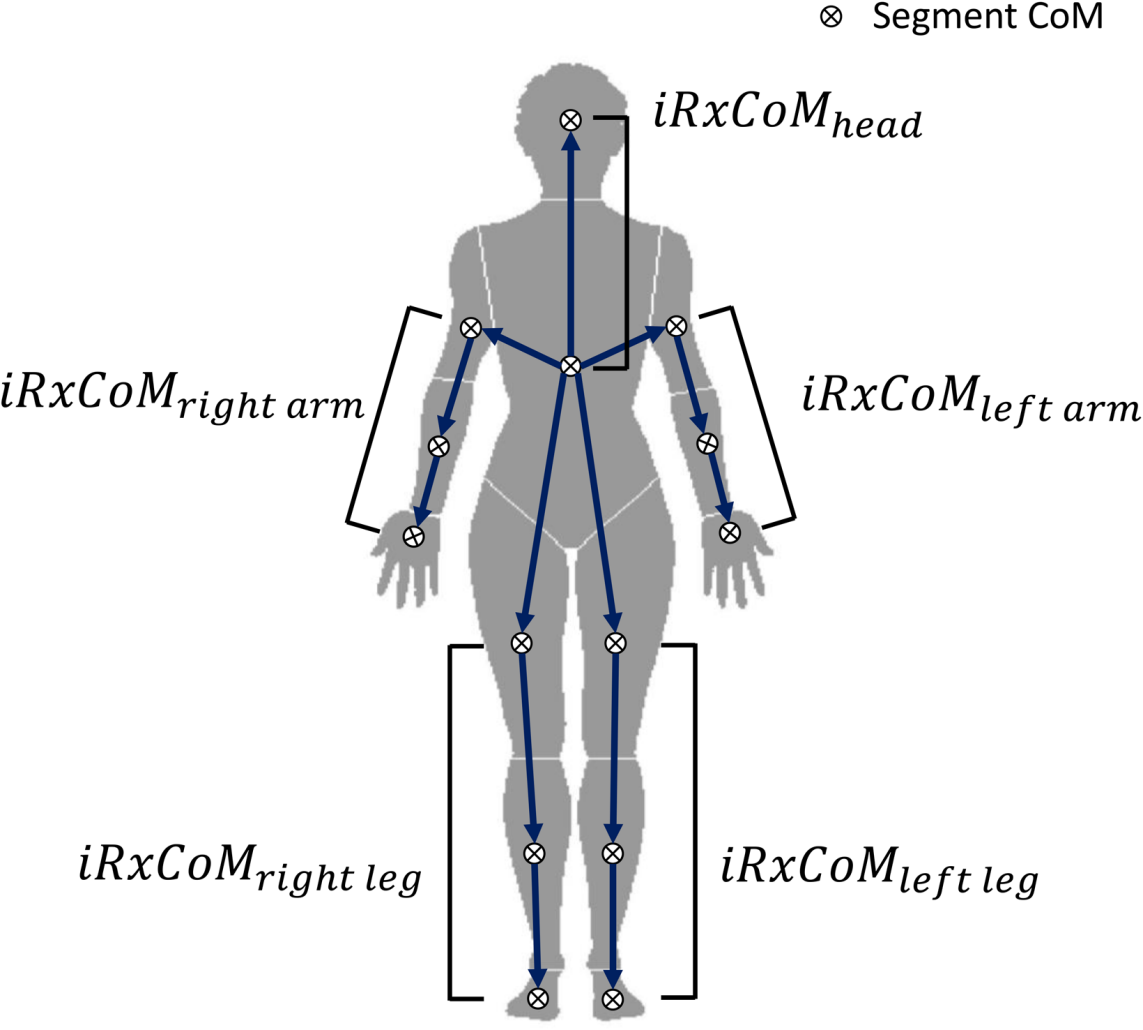
Relative individual segmental extrapolated center of mass ($iRx\mathrm{CoM}$) in human skeletal system. Five body parts (right and left arms, right and left legs, and head) from the trunk segment were separated. In each body part, $iRx\mathrm{CoM}$ was calculated as individual segmental CoM positions based on the proximal segmental CoM. Drawn using Adobe Illustrator CS6 (www.adobe.com) and Microsoft Office 2016 (www.microsoft.com).

Note that the trunk segment is a base segment, indicating ${ix\mathrm{CoM}}_{trunk}\left[t\right]={iRx\mathrm{CoM}}_{trunk}\left[t\right]$, and five body parts (right and left arms with upper arms, forearms, and hands, right and left legs with thighs, shanks, and feet, and head) connected to the trunk segment as serial chains were separated for this calculation (Fig. 2) in order to extract independent variables from individual segment CoMs. Thus, the task equation of the performance variable as a function of the elemental variables is as follows (Eq. 7).

7 $$x{\text{CoM}}\left[ t \right] = f\left( {iRx{\text{CoM}}\left[ t \right]} \right)$$

Using the Jacobian matrix (**J**), the task equation of $x\mathrm{CoM}\left[t\right]$ was derived in terms of ${\varvec{i}}{\varvec{R}}{\varvec{x}}\mathbf{C}\mathbf{o}\mathbf{M}\left[t\right]$ as follows (Eq. 8):

8 $$x\mathrm{CoM}\left[t\right]=\mathbf{J}\cdot {\varvec{i}}{\varvec{R}}{\varvec{x}}\mathbf{C}\mathbf{o}\mathbf{M}\left[t\right]=\left[\begin{array}{cc}\begin{array}{ccc}\frac{\partial x\mathrm{CoM}}{\partial {iRx\mathrm{CoM}}_{1}}& \frac{\partial x\mathrm{CoM}}{\partial {iRx\mathrm{CoM}}_{2}}& \cdots \end{array}& \frac{\partial x\mathrm{CoM}}{\partial {iRx\mathrm{CoM}}_{n}}\end{array}\right]\left[\begin{array}{c}\begin{array}{c}{iRx\mathrm{CoM}}_{1}\left[t\right]\\ {iRx\mathrm{CoM}}_{2}\left[t\right]\\ \vdots \end{array}\\ {iRx\mathrm{CoM}}_{n}\left[t\right]\end{array}\right]$$

Here, the Jacobian matrix is time invariant, containing a constant value in each element which usually differs from the Jacobian of joint-angle non-linear kinematics used for a linear approximation. The Jacobian matrix is identical between AP direction and ML direction. The null space of the Jacobian, spanned by the basis vector, $${\varvec{\varepsilon}}$$, is the subspace of $${\varvec{i}}{\varvec{R}}{\varvec{x}}\mathbf{C}\mathbf{o}\mathbf{M}\left[t\right]$$ that does not affect changes in $$x\mathrm{CoM}\left[t\right]$$ (i.e. the task-irrelevant space). Using the UCM analysis, $${\varvec{i}}{\varvec{R}}{\varvec{x}}\mathbf{C}\mathbf{o}\mathbf{M}\left[t\right]$$ is decomposed into task-relevant and task-irrelevant components. The dimension of $${\varvec{\varepsilon}}$$ is $$n-d$$ where $$n$$ is the number of elemental variables ($$n$$ =14) and *d* is the number of performance variable ($$d=1)$$. Note that we performed the analysis for two consecutive phases: the first phase (Phase I) was from the onset of perturbation to the moment when the whole-body xCoM in AP passed outside its base of support (BoS) of the non-stepping foot, and the second phase (Phase II) was from the moment when the xCoM in AP passed outside the BoS to the time of foot–ground contact of the stepping foot (Fig. 1B). Since the one forward step occurs in AP, two phases were determined when the whole-body xCoM in AP is greater or lower than the actual toe position in AP. The actual toe position was estimated as a position of the tip of the toe by scaling the vector from heel marker to toe marker with subject’s actual foot size.

The task-irrelevant component (${{\varvec{i}}{\varvec{R}}{\varvec{x}}\mathbf{C}\mathbf{o}\mathbf{M}}_{{\varvec{T}}{\varvec{I}}{\varvec{R}}}\left[t\right]$**)** was obtained by projecting ${\varvec{i}}{\varvec{R}}{\varvec{x}}\mathbf{C}\mathbf{o}\mathbf{M}\left[t\right]$ onto the null space (or the task-irrelevant space) of the Jacobian (Eq. 9).

9 $${{\varvec{i}}{\varvec{R}}{\varvec{x}}\mathbf{C}\mathbf{o}\mathbf{M}}_{{\varvec{T}}{\varvec{I}}{\varvec{R}}}\left[t\right]={\varvec{\varepsilon}}\cdot {{\varvec{\varepsilon}}}^{{\varvec{T}}}\cdot \left({\varvec{i}}{\varvec{R}}{\varvec{x}}\mathbf{C}\mathbf{o}\mathbf{M}\left[t\right]-{\varvec{i}}{\varvec{R}}{\varvec{x}}\mathbf{C}\mathbf{o}\mathbf{M}\left[0\right]\right)$$

The task-relevant component (${{\varvec{i}}{\varvec{R}}{\varvec{x}}\mathbf{C}\mathbf{o}\mathbf{M}}_{{\varvec{T}}{\varvec{R}}}\left(t\right)$), was obtained by projecting ${\varvec{i}}{\varvec{R}}{\varvec{x}}\mathbf{C}\mathbf{o}\mathbf{M}$ onto the range space (perpendicular to the null space), and computed as follows (Eq. 10):

10 $${{\varvec{i}}{\varvec{R}}{\varvec{x}}\mathbf{C}\mathbf{o}\mathbf{M}}_{{\varvec{T}}{\varvec{R}}}\left[t\right]=\left({\varvec{i}}{\varvec{R}}{\varvec{x}}\mathbf{C}\mathbf{o}\mathbf{M}\left[t\right]-{\varvec{i}}{\varvec{R}}{\varvec{x}}\mathbf{C}\mathbf{o}\mathbf{M}\left[0\right]\right)-{{\varvec{i}}{\varvec{R}}{\varvec{x}}\mathbf{C}\mathbf{o}\mathbf{M}}_{{\varvec{T}}{\varvec{I}}{\varvec{R}}}\left[t\right]$$

where in Phase I, ${\varvec{i}}{\varvec{R}}{\varvec{x}}\mathbf{C}\mathbf{o}\mathbf{M}\left[0\right]$ is a vector at the onset of perturbation, and in Phase II, ${\varvec{i}}{\varvec{R}}{\varvec{x}}\mathbf{C}\mathbf{o}\mathbf{M}\left[0\right]$ is at the time of $x\mathrm{CoM}$ passing outside the forward edge of BoS of CS.

A mean squared deviation ($MSD$) , the sum of individual segment $MSD$ s (Eqs. 11 and 12) of the projected deviations ($iMSD$) (Eqs. 13 and 14), was computed in both the task-relevant (${MSD}_{TR}$) and the task-irrelevant spaces (${MSD}_{TIR}$) as follows:

11 $${MSD}_{TR}=\sum_{i=1}^{n}{{iMSD}_{TRi}}$$

12 $${MSD}_{TIR}=\sum_{i=1}^{n}{{iMSD}_{TIR}}_{i}$$

where

13 $${{iMSD}_{TR}}_{i}=\frac{1}{N}\sum_{t=1}^{N}{\left({{iRx\mathrm{CoM}}_{TR}}_{i}\left[t\right]\right)}^{2}$$

14 $${{iMSD}_{TIR}}_{i}=\frac{1}{N}\sum_{t=1}^{N}{\left({{iRx\mathrm{CoM}}_{TIR}}_{i}\left[t\right]\right)}^{2}$$

where N is the number of samples.

In order to quantify synergistic multi-segmental coordination patterns of dynamic postural control, the index of synergy, $SYN$ (Eq. 15), was computed.

15 $$SYN= \left(\frac{{MSD}_{TIR}}{\left(n-d\right)}-\frac{{MSD}_{TR}}{d}\right)\frac{1}{{MSD}^{TIR}+{MSD}^{TR}}$$

where $$\left(n-d\right)$$ and $$d$$ are DoFs in task-irrelevant space and task-relevant space, respectively. Note that $$n$$ is the number of elemental variables ($$n$$ =14) and *d* is the number of performance variable ($$d=1)$$.

Similar to ${{iMSD}_{TR}}_{i}$ and ${{iMSD}_{TIR}}_{i}$ above, we further computed the *i*th segment contribution to $SYN$, ${iSYN}_{i}$, which is mathematically equivalent to the amount of $SYN$ projected onto each individual segmental dimension (Eq. 16).

16 $$iSYN_{i} = \frac{{iMSD_{TIRi} - iMSD_{TRi} }}{{ iMSD_{TIRi} + iMSD_{TRi} }}$$

$SYN$ quantifies to what extent the element variables (i.e. segment CoM, ${\varvec{i}}{\varvec{R}}{\varvec{x}}\mathbf{C}\mathbf{o}\mathbf{M}$) are coordinated to stabilize the performance variable (i.e. whole-body CoM, $x\mathrm{CoM}$). Greater $SYN$ indicates that the element variables (i.e. individual segment CoMs) are coordinated better for the stabilization of the performance variable (i.e. whole-body CoM). Note that ${{iMSD}_{TR}}_{i}$, ${{iMSD}_{TIR}}_{i}$ and ${iSYN}_{i}$ are the *i*^th^ segment contribution to ${MSD}_{TR}$, ${MSD}_{TIR}$, and $SYN$, respectively. Geometrically, ${{iMSD}_{TR}}_{i}$, ${{iMSD}_{TIR}}_{i}$ and ${iSYN}_{i}$ indicate that the amounts of ${MSD}_{TR}$, ${MSD}_{TIR}$ and *SYN* projected onto individual segmental dimension, respectively.

### Statistical analyses

A two-way repeated measures ANOVA with two factors Group (2 levels: dancers vs non-dancers) and Segment (14 levels: head, trunk, and left & right upper arms, left & right forearms, left & right hands, left & right thighs, left & right shanks, and left & right feet) were performed along with a post-hoc test with Bonferroni correction to test the differences of segments between groups. Separate ANOVAs were conducted for the two phases and two dimensions (AP and ML). The level of statistical significance was set at p = 0.05. Standard descriptive statistics were used; the data are presented as means ± standard errors.

## Results

First, we checked which foot participants used for the forward step. While all participants took one forward step, the stepping foot differed between participants: 7 participants with the left foot and 13 participants with the right foot. The stepping foot was designated as the ipsilateral side (IS) and the non-stepping foot as the contralateral side (CS) for the purpose of reporting results. In the ML direction, dancers did not differ from non-dancers in either Phase I (the $x\mathrm{CoM}$ is inside of the BoS) or Phase II (when the $x\mathrm{CoM}$ is outside the BoS). In the AP direction, none of the dependent variables, ${MSD}^{TR}$, ${MSD}^{TIR}$, $SYN$, ${iMSD}^{TR}$, ${iMSD}^{TIR}$, and $iSYN$ differed between dancers and non-dancers in Phase I. These results indicate that the postural control responses to the perturbation were similar between dancers and non-dancers in Phase I.

**Figure 3:**
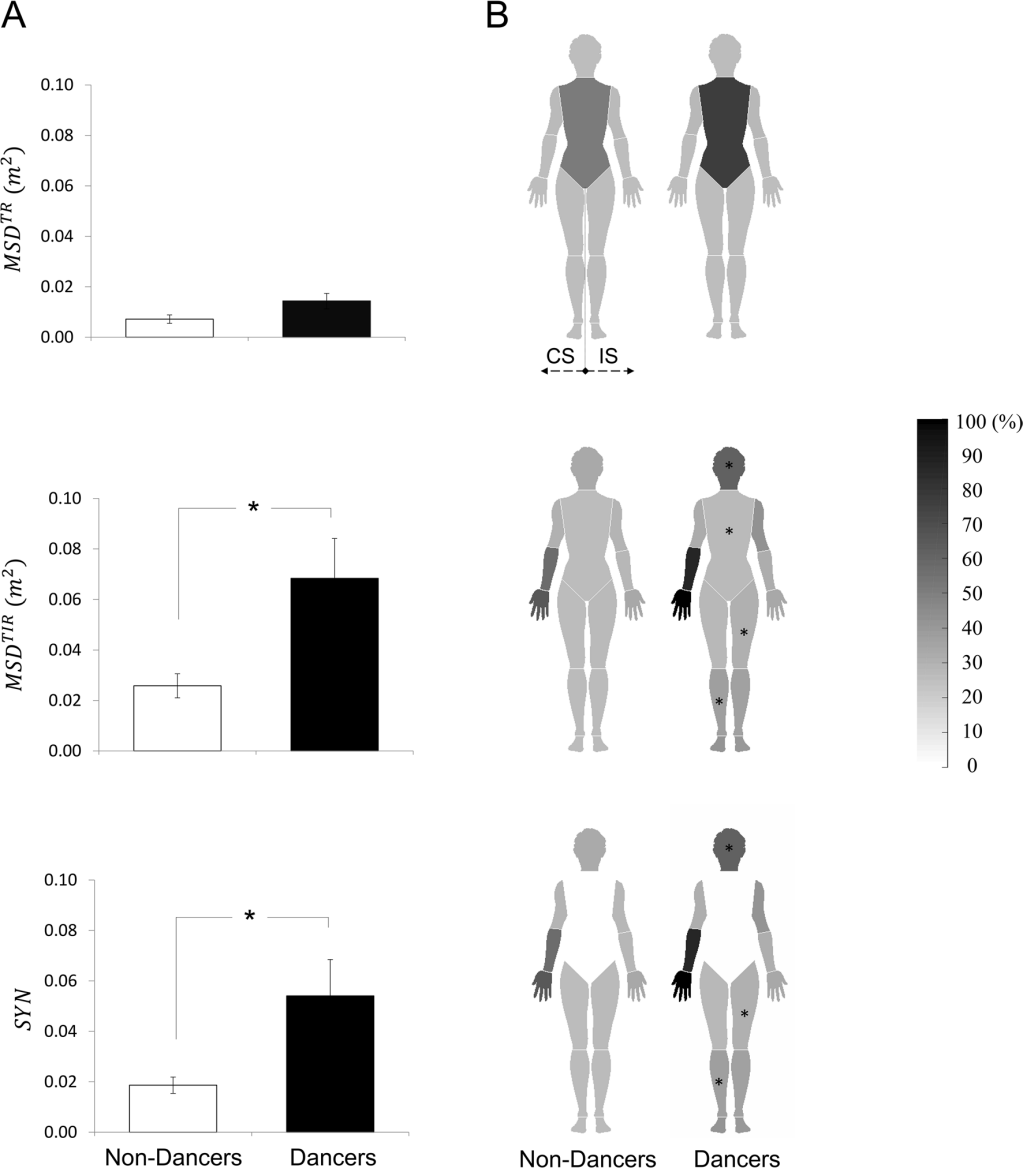
Task-relevant mean squared deviation ($${MSD}^{TR}$$), task-irrelevant mean squared deviation ($${MSD}^{TIR})$$, and index of synergy ($$SYN$$) for dancers and non-dancers. mean $${MSD}^{TR}$$, $${MSD}^{TIR}$$ and $$SYN$$ for dancers (white) and non-dancers (black) are shown in panel A. Error bars represent a standard error across subjects. Percentage of individual segment contributions to each of $${MSD}^{TR}$$, $${MSD}^{TIR}$$ and $$SYN$$ are shown as multi-segmental human geometry with the contralateral side (CS) and ipsilateral side (IS) in panel B. There exists a significant interaction Group X Segments on $${MSD}^{TIR}$$ and $$SYN$$. Post hoc pairwise comparisons revealed that 1) $${MSD}^{TIR}$$ at head, trunk, CS thigh, and IS shank, and 2) $$SYN$$ at head, CS thigh, and IS shank in dancers were significantly greater as compared to non-dancers. The asterisk indicates a significant difference (*p < 0.05) between dancers and non-dancers. Drawn using Adobe Illustrator CS6 (www.adobe.com) and Microsoft Office 2016 (www.microsoft.com).

In Phase II, ${MSD}^{TIR}$ was significantly greater in dancers compared to non-dancers (F_1,18_ = 5.986; p = 0.025) (Fig. 3A), which was supported by a significant interaction Group X Segment (F_1,18_ = 2.399; p = 0.005). Individual segment comparisons revealed significant differences of ${iMSD}^{TIR}$ at head (p = 0.013), trunk (p = 0.044), CS thigh (p = 0.021), and IS shank (p = 0.011) between dancers and non-dancers, while no difference was found at CS shank (p = 0.083), CS foot (p = 0.961), IS thigh (p = 0.302), IS foot (p = 0.067), CS upper arm (p = 0.076), CS forearm (p = 0.183), CS hand (p = 0.281), IS upper arm (p = 0.117), IS forearm (p = 0.245), and IS hand (p = 0.268). Thus, the greater ${MSD}^{TIR}$ of Dancers was mainly due to the greater values on ${MSD}^{TIR}$ at head, trunk, CS thigh, and IS shank (Fig. 3B). However, there was no significant difference in ${MSD}^{TR}$ between dancers and non-dancers (F_1,18_ = 3.700; p = 0.070), along with no differences in individual segments ${iMSD}^{TR}$. In terms of synergy, dancers showed greater $SYN$ than non-dancers (F_1,18_ = 5.152; p = 0.036) (Fig. 3A). These results were supported by a significant interaction Group × Segment (F_1,18_ = 3.069; p < 0.001). The pairwise comparisons of individual segments revealed significant differences of $SYN$ at head (p = 0.013), CS thigh (p = 0.023) and IS shank (p = 0.011) between dancers and non-dancers, while no difference was found at trunk (p = 0.075), CS shank (p = 0.084), IS thigh (p = 0.597), CS upper arm (p = 0.081), CS forearm (p = 0.184), CS hand (p = 0.281), IS upper arm (p = 0.130), IS forearm (p = 0.245), or IS hand (p = 0.268) (Fig. 3B). Thus, the greater $SYN$ demonstrated by dancers was mainly due to the higher values on $SYN$ at head, CS thigh, and IS shank.

## Discussions

We investigated the dynamic postural control strategies during perturbation-evoked stepping between cohorts of dancers and non-dancers. In the current study, we present a new approach to investigate dynamic postural control for multiple segment coordination. Our analysis was performed in two phases: during the stable phase (i.e. Phase I) where the whole-body CoM was within BoS and during the unstable phase (i.e. Phase II) where the whole-body CoM was outside of BoS. In ML movement, we found no significant difference between dancers and non-dancers in either Phase I or Phase II. However, in AP movement, as hypothesized, we found that dancers had significantly greater index of synergy, $SYN$, than non-dancers in Phase II, although we found no significant difference between dancers and non-dancers for Phase I.

The findings that dancers had greater motor synergy (i.e. increased *SYN*) implies that dancers exhibited different postural control strategies compared to non-dancers while exploiting redundant DoFs (i.e. relative segments, $iRx\mathrm{CoM}$) of the multi-linked kinematic body. In the current study, the greater synergy exhibited by dancers was attributed to greater task-irrelevant deviation, ${MSD}^{TIR}$ compared to non-dancers. The findings of greater ${MSD}^{TIR}$ in dancers indicate that dancers used more variability of individual segments that resulted in a consistent whole-body CoM position. On the other hand, task relevant deviation, ${MSD}^{TR}$, did not differ between groups, which suggests that both groups had similar trajectories of the whole-body CoM position. Although some studies reported that dancers have superior performance on their balance control as compared to non-dancers^29,30^, our results do not directly indicate dancers have superior balance performance because of the same ${MSD}^{TR}$. Consistent with our findings of a greater synergy index, previous studies reported that experts in surgical robot operation^31^, stone knapping^32^, and cello bowing^33^ use a different motor strategy while exploiting redundancy in the motor system by having greater task-irrelevant variability without deteriorating performance. According to the principle of non-individualized control, multiple independent motor effectors (e.g. muscles, joints, or segments) are not controlled individually by the CNS, but are rather united as a task-specific organization^34^. In support of the principle, our finding implies that dynamic control of the whole-body CoM position in dancers is achieved through the use of motor equivalent combinations of multiple segments, rather than reducing the variability of individual segments CoM positions.

Our analysis for individual segment contributions revealed that ${MSD}^{TIR}$ at head, trunk, CS thigh, and IS shank in dancers are greater compared to non-dancers. The greater ${MSD}^{TIR}$ in dancers is attributed to a greater $SYN$ at head, CS thigh, and IS shank segments. Our results indicate that dancers have superior postural coordination ability by allowing more variations in multi-segment configurations especially at head, trunk, CS thigh, and IS shank segments to stabilize whole-body CoM position. Our results are consistent with previous findings. Many previous studies on upper extremity movements have indicated that controlling head and trunk segments is critical for postural control by demonstrating that the head was well stabilized during walking^35–37^. It has been suggested that the head stability is achieved in compensatory mechanisms of head-trunk coordination^38^. In terms of the lower extremities, importance of hip and ankle joints for postural control has been reported^39,40^. Riemann, et al.^39^ found that the ankle joint plays an important role in corrective action during a single-leg stance. Vlutters et al.^40^ reported that the hip joint acts primarily for foot placement adjustments of the swing leg, which is often considered as an important strategy for postural control during walking. These strategies regarding the ankle in the stance leg and the hip in the swing leg are supported by the findings of greater ${MSD}^{TIR}$ shown in CS thigh and IS shank in dancers in our study.

The current study, for the first time, provided evidence of dancers possessing superior coordination of body segments and ability for dynamic postural control of their multi-segment body. Based on previous studies^19,41^, it has been generally accepted that dancers have superior postural control ability compared to novices. The present findings for superior postural control ability during the unstable phase are consistent with findings in previous studies on dancers^30,42,43^. For instance, superior balance control of dancers as compared to non-dancers was found in more challenging motor tasks such as one-legged stance^30,42^ and balance on a boat^43^. In addition, Krityakiarana and Jongkamonwiwat^44^ found that dancers exhibited better performance in maintaining postural stability than non-dancers during additional multitask conditions. However, to our knowledge, superior coordination ability in dancers in the context of motor synergy has not been reported previously. Our findings of a greater task-irrelevant variability in dancers indicate greater flexibility in utilizing DoFs to accomplish the motor task. Previous studies suggested that such flexibility would be beneficial in dealing with challenges in external and internal constraints, such as unexpected perturbations^14^, fatigue^15^, and performing secondary tasks^16^. Consistent with previous studies, our findings suggest that a long-term specialized dance training can improve the CNS’s flexibility for exploitation of the redundant DoFs in the whole-body system.

The idea behind motor synergy under the framework of the UCM hypothesis is that the CNS utilizes elemental variables or DoF in a way to ensure stability of a performance variable, which typically results in task-specific organization of the elemental variables^45–47^. In this study, we presented a new approach to quantify kinematic synergy of multi-segments. For the approach, we introduced the relative individual segment CoMs as element variables in the UCM analysis for the consideration of mathematical independency. Even though there are many studies on postural control in various behaviors such as standing^17,18^ and walking^23–25^, these studies have focused on dynamic stability of the whole-body CoM in relation to its base of support. As a consequence, these studies did not investigate the CNS control strategies over multi-segmental synergistic patterns.

## Limitations

In our UCM analysis, segment CoM does not contain orientation information, which may play a role in contribution to postural coordination especially during turning or twisting motions even though it may not be critical in the perturbation-induced stepping movement in the current study design. Even though previous studies using UCM analysis investigated joint angles in relation to whole-body CoM control^5,48^, the quantification of whole-body CoM using multi-segment CoMs proposed in the current study may provide a more accurate measurement than using joint angles: both approaches require estimations of inertia properties such as segment mass and moment of inertia which propagate errors throughout the calculations for whole-body CoM estimation. However, joint angle calculations require additional estimation of joint centers, which may result in inflated uncertainty in calculation of the whole-body CoM.

## Conclusion

In the current study, we presented a new approach to quantify kinematic synergy within-trials during postural control. Few studies exist in which researchers investigated how the CNS manages postural control of multiple body segments^6,49^. However, those studies have quantified motor synergy of multi-joint kinematics in relation to the whole-body CoM in a static state. Thus, those studies might not have fully captured the CNS control mechanisms in dynamic behaviors. In addition, many previous studies on motor synergy of dynamic movements analyzed trial-to-trial variability using the Jacobian matrix to linearize non-linear task equation in terms of joint angles^6,28,47^. However, that approach to within-trial analysis was not appropriate because the Jacobian matrix is a linearization method for small deviations around the average trajectories. In the current study, we quantified mean squared deviations from initial posture at each phase rather than quantifying trial-to-trial variances of mean CoM trajectory quantified by previous UCM studies on whole-body movements. Given that quantification of moment-to-moment (or within-trial) variability and trial-to-trial variability can provide distinct features of the CNS’s control mechanism, dancers’ superior coordination ability reflected in trial-to-trial movement variability warrants a future investigation.
